# Growth but Not Photosynthesis Response of a Host Plant to Infection by a Holoparasitic Plant Depends on Nitrogen Supply

**DOI:** 10.1371/journal.pone.0075555

**Published:** 2013-10-07

**Authors:** Hao Shen, Shu-Jun Xu, Lan Hong, Zhang-Ming Wang, Wan-Hui Ye

**Affiliations:** 1 Key Laboratory of Vegetation Restoration and Management of Degraded Ecosystems, South China Botanical Garden, Chinese Academy of Sciences, Guangzhou, Guangdong, PR China; 2 College of Life Sciences, University of Chinese Academy of Sciences, Beijing, PR China; 3 College of Horticulture and Landscape Architecture, Zhongkai University of Agriculture and Engineering, Guangzhou, Guangdong, PR China; CNRS, Université de Bourgogne, France

## Abstract

Parasitic plants can adversely influence the growth of their hosts by removing resources and by affecting photosynthesis. Such negative effects depend on resource availability. However, at varied resource levels, to what extent the negative effects on growth are attributed to the effects on photosynthesis has not been well elucidated. Here, we examined the influence of nitrogen supply on the growth and photosynthesis responses of the host plant *Mikania micrantha* to infection by the holoparasite *Cuscuta campestris* by focusing on the interaction of nitrogen and infection. *Mikania micrantha* plants fertilized at 0.2, 1 and 5 mM nitrate were grown with and without *C. campestris* infection. We observed that the infection significantly reduced *M. micrantha* growth at each nitrate fertilization and more severely at low than at high nitrate. Such alleviation at high nitrate was largely attributed to a stronger influence of infection on root biomass at low than at high nitrate fertilization. However, although *C. campestris* altered allometry and inhibited host photosynthesis, the magnitude of the effects was independent of nitrate fertilizations. The infection reduced light saturation point, net photosynthesis at saturating irradiances, apparent quantum yield, CO_2_ saturated rate of photosynthesis, carboxylation efficiency, the maximum carboxylation rate of Rubisco, and maximum light-saturated rate of electron transport_,_ and increased light compensation point in host leaves similarly across nitrate levels, corresponding to a similar magnitude of negative effects of the parasite on host leaf soluble protein and Rubisco concentrations, photosynthetic nitrogen use efficiency and stomatal conductance across nitrate concentrations. Thus, the more severe inhibition in host growth at low than at high nitrate supplies cannot be attributed to a greater parasite-induced reduction in host photosynthesis, but the result of a higher proportion of host resources transferred to the parasite at low than at high nitrate levels.

## Introduction

Parasitic plants are a taxonomically diverse group of organisms that obtain some or all of their nutrients and other resources, such as water, carbon and phytohormones, from their host plants via haustoria [1]. Interactions between them and their hosts are one of the key research topics in parasitic plant biology [2], [3]. Press *et al.* [1] indicated that the extent to which parasites compete with their hosts for nutrients depends on the relative sink strength and the degree of autotrophy of the parasites. In hemiparasitic plants, nutrient transfer and resource acquisition from the hosts are facilitated by the parasite maintaining high transpiration rates, high leaf conductance and low water potentials, and in holoparasitic plants, by high osmotic potentials [3]. Furthermore, parasitic plants can affect the photosynthesis of their hosts at the leaf and/or whole plant level [4]. These processes can adversely affect the hosts, and such negative effects depend on resource availability: they might be negligible when resources are abundant but when resources are limiting they can be severe, ranging from reduction of growth and development to death of the hosts [3].

The influence of nitrogen on host-parasite associations has been investigated in the economically important root hemiparasite *Striga hermonthica* [5], [6] and the stem holoparasite *Cuscuta reflexa* [7], [8]. *Striga hermonthica*-infected C_4_ sorghum had lower rates of photosynthesis than uninfected plants, but the difference in both growth and photosynthesis between uninfected and infected sorghum plants was lower or even negligible when high nitrogen concentrations were supplied [5]. In contrast, high nitrogen supply did not result in an alleviation of the effects of the parasite on the host C_3_ rice to the same degree that *S. hermonthica* did on the sorghum host, as reflected by similar growth and photosynthesis in uninfected and infected plants at high nitrogen supply [6].

Among the species in *Cuscuta* (Convolvulaceae), nitrogen relations in the parasitic associations of *C. reflexa* and its leguminous or non-leguminous hosts have been studied [7]–[9]. Modelling the solute transfer between *C. reflexa* and its leguminous host *Lupinus albus* [9] indicated that the massive demand of the parasite led to resource losses of the host, particularly nitrogen from leaves and roots. As a result of such highly competitive sink activity of the parasite, net photosynthesis of *L. albus* appeared to be stimulated. However, *C. reflexa* infection increased tissue nitrogen levels in the non-N_2_-fixing hosts *Ricinus communis* [8] and *Coleus blumei* [7]. Growth and development of *C. reflexa* were restricted similarly with those of the hosts when fed with different concentrations of nitrate, suggesting a fine tuning of the parasite sink strength with the source capacity of both hosts [7], [8]. In these associations, *C. reflexa* led to a substantial sink-dependent stimulation of the host’s photosynthesis and, under N-limiting conditions, to an increase in the host’s tissue nitrogen concentrations. The reason for the different effects of *C. reflexa* on the symbiotically N-fed *L. albus* and on nitrate-fed *R. communis* and *C. blumei* was attributed to the overriding competition between *C. reflexa* and *L. albus* in the tripartite association *L. albus*-Rhizobium-*C. reflexa*, whilst this additional factor was absent in the associations with *R. communis* and *C. blumei* [7], [8]. Although the holoparasite *C. reflexa* substantially decreased growth of both *R. communis* [8] and *C. blumei* [7] regardless of nitrate supply, the inhibition in growth of infected *R. communis* was exacerbated at low N supply, but in contrast, the inhibition in growth of infected *C. blumei* was similar at low and high N supply.

In our previous studies, we investigated the influence of another *Cuscuta* species, *C. campestris,* on growth, biomass allocation and photosynthesis of an invasive weed, *Mikania micrantha* H.B.K. (Asteraceae). We found different growth and photosynthesis influence patterns from those of *C. reflexa*. *Cuscuta campestris* significantly reduced the total biomass, changed the biomass allocation patterns and completely inhibited the flowering of *M. micrantha* plants [10]. In addition to direct resource capture by *C. campestris,* the parasite also reduced the stomatal conductance, and carboxylation and light use efficiencies of the host, resulting in reduced growth of infected plants [11]. We also observed that the total biomass of the parasite plus its host was significantly less than that of uninfected hosts [10], and the parasite suppressed photosynthesis of the hosts [11]. However, Jeschke and Hilpert [8] and Jeschke *et al.* [7] observed that the total biomass of *C. reflexa* plus its hosts was similar to that of the uninfected and *C. reflexa* led to a sink-dependent stimulation of host photosynthesis. Thus, it is of interest to study if the nitrogen relations are also different between *C. campestris*-host and *C. reflexa*-host associations.

In the present project we investigated the nitrogen relations in the *M. micrantha*-*C. campestris* host-parasite association by focusing on the interaction of nitrogen and infection. We hypothesized that both growth and photosynthesis responses in *M. micrantha* to *C. campestris* infection would be more affected by parasitism at low than high nitrogen supply.

## Materials and Methods

### Study Species

*Mikania micrantha* H.B.K. is a fast-growing climbing perennial vine of the family Asteraceae, native to Central and South America [12]. In its palaeotropic exotic range, it is a notorious invasive weed, severely damaging forestry and plantation crops [13]. In South China, it grows in poor to fertile soils with total nitrogen 0.14–1.62 g kg^–1^ [13]. In the field, the generalist stem parasite *Cuscuta campestris* Yuncker infects *M. micrantha* and it has been one of the most effective means of biologically controlling *M. micrantha* in South China [10], [13]. *Cuscuta campestris* is the most widespread species in the genus and the only parasitic weed of North America that has spread to the Old World [14]. It is a holoparasite and draws all nutrients from its host. It is a very powerful sink for host photosynthates, severely suppressing host growth, preventing flowering and fruiting, and even resulting in host death [10], [14]. It can infect many herbaceous plants and results in damage to horticultural and agricultural crops, and it is the worst pest of alfalfa and other legumes [14].

### Plant Culture and Growth Conditions

The experiment was carried out during the July 2011–January 2012 growing season in an unheated greenhouse with natural light at the same field station of South China Botanical Garden as in our previous study [10]. On 26 July 2011, whole *M. micrantha* plants were collected from a *M. micrantha* population near the station. Two-node segments, similar in size, were obtained from the middle of the stems. The segments were planted in containers filled with washed moist sand, with the low nodes buried below and the upper about 5 cm above the sand surface. The upper nodes began to sprout 5 days later. On 20 August, 90 healthy sprouts about 20 cm long were transplanted into 8.36 L pots filled with washed moist sand, one per pot, and the pots were placed in the glasshouse with a temperatures range 12–28°C, mean 17.8°C, and relative humidity range 50–90%, mean 70% during August 2011–January 2012. The plants were watered twice daily at 06∶00 h and 18∶00 with distilled water during the first week after transplanting. From then on to the end of the experiment, they were watered at 06∶00 h with distilled water and at 18∶00 h with modified Hoagland solutions containing 0.2, 1 or 5 mM nitrate with 200 ml per pot and 30 pots per nitrate concentration. The pots were thoroughly rinsed with water once a week.

On 7 October when the *M. micrantha* plants had been treated with nitrate for 41 days, half of them within each nitrate treatment were randomly chosen and inoculated with *C. campestris* filaments about 5 cm in length, one per plant, and the rest were left as control. To ensure simultaneous attachment, excised and previously twined shoot cuttings of *C. campestris* were allowed to attach to the lowest two *M. micrantha* stem internodes. By 14 October, all the inoculated *M. micrantha* plants had become infected with *C. campestris* stems as indicated by renewed vigorous growth of the filaments. Thus, this day was considered day 0 after parasitization (DAP). To prevent *M. micrantha* from climbing from one pot to another, a bamboo cane was placed vertically in each pot for *M. micrantha* to climb on. The experiment ended on 14 January 2012, 90 DAP or 172 days after planting, when the uninfected *M. micrantha* plants fertilized at 5 mM nitrate were in full bloom.

### Growth Measurements and Observations

During the experiment, both destructive and nondestructive measurements of growth were made. Height from the base of the stem to the apex of the shoot and number of visible leaves per *M. micrantha* plant were recorded on 0, 15, 40, 60, 90 DAP. Flowering times of *M. micrantha* and *C. campestris* plants were also recorded.

*Mikania micrantha* plants on 0 DAP, and the uninfected and infected and parasite plants on 90 DAP were randomly sampled and harvested, five per treatment. We measured the leaf area using a LI-3000C portable laser area meter (LI-COR Inc., Lincoln, NE, USA), removed the dead material and counted the number of dead leaves of the sampled *M. micrantha* plants, but the number of dead leaves was not used in the growth analyses. We separated the living parts of the sampled plants into stems, leaves, reproductive organs (if present) and roots. Roots were soaked in tap water, washed and separated carefully in running water over a 2-mm mesh sieve. Stems, tendrils and reproductive organs of *C. campestris* were carefully dissected from stems and leaves of *M. micrantha* plants.

All plant material was oven dried at 70°C until constant weights were achieved, and they were used to obtain tissue C and N concentrations and dry weights. For the *M. micrantha* plants harvested on 90 DAP, specific leaf area (SLA, the ratio of leaf area to dry mass) and shoot-to-root dry weight ratio (S/R), relative growth rate (RGR, the dry weight increase per plant per day), leaf area ratio (LAR, the ratio of leaf area to dry weight per plant) and unit leaf rate (ULR, dry weight production per m^2^ leaf area per day) were calculated according to Hunt and Parsons [15].

### Measurements of Photosynthesis

*In situ* gas exchange measurements were made on *M. micrantha* leaves using a LI-6400 portable photosynthesis system with a standard 6 cm^2^ leaf chamber (LI-COR Inc., Lincoln, NE, USA) on 30 and 80 DAP, at around 10∶00 h, and photosynthetic parameters were calculated based on von Caemmerer and Farquhar [16]. To ensure that leaves measured were similar in age and developmental stage, only the youngest fully expanded mature sun leaves were sampled, one leaf per plant, from five randomly selected *M. micrantha* plants per treatment. Conditions inside the leaf chamber during the measurements were controlled as follows. Irradiance was provided by an integrated red-blue light-emitting diode source (model 6400-02B, LI-COR, Inc.) at photosynthetic photon flux density (PPFD) of 1000 µmol photons m^−2^ s^−1^ except for the light response study, CO_2_ concentration (*C*_a_) was controlled at 360 µmol mol^−1^ with a CO_2_ mixer except for the leaf internal CO_2_ concentration (*C*_i_) response study, flow rate was set at 500 µmol s^−1^, and leaf temperature (*T*_l_) was controlled at 20°C on 80 DAP and at 30°C on 30 DAP. Net photosynthetic rate (*P*_n_), stomatal conductance (*g*_s_, mol H_2_O m^–2^ s^–1^), rate of transpiration (*E*, mmol H_2_O m^−2^ s^−1^), intercellular CO_2_ concentration (*C*_i_), *C*_a_, air temperature (*T*_a_), *T*_l_, air relative humidity (RH), and PPFD were recorded after equilibration to a steady state with a coefficient of variation ≤1% at each measurement had been reached. Water use efficiency (WUE, µmol CO_2_ mmol H_2_O^–1^) was calculated as *P*_n_/*E* for each measurement. Methods and conditions used to obtain photosynthesis light and *C*_i_ response curves were the same as described in the above paragraph unless specified otherwise.

### Determination of Chlorophyll and Carotenoid Concentrations

Leaf chlorophyll concentrations were measured on the leaves used for the measurements of photosynthesis. Leaf pigments were extracted from about 70 mg of leaf sample put in 10 mL 80% acetone for 72 hours in the dark, and carotenoid and chlorophyll *a* and *b* concentrations were determined spectrophotometrically at 663, 645 and 470 nm according to Arnon [17].

### Light Response Curves

To construct light response curves, on two clear days, 80–81 DAP, photosynthesis measurements were made between 08∶00 h and 11∶00 h. Leaf temperature in the leaf chamber was maintained at 20°C. When a leaf in the chamber had acclimated to a PPFD of 500 µmol photons m^−2^s^−1^ for 20 min, photosynthesis measurements were taken at PPFD in the following order: 500, 800, 1000, 1500, 1800, 2000, 200, 100, 50, 20, 0 µmol photons m^−2^s^−1^. For each measurement, apparent quantum yield (Φ, mol CO_2_ mol^−1^ photons), dark respiration rate (*R*_d_, µmol CO_2_ m^−2^s^−1^) and light compensation point (LCP) were obtained by linear regression using data obtained at PPFD of 0, 20 and 50 µmol photons m^−2^s^−1^ [18]. The entire photosynthetic light response curves were fitted using Photosynthesis Work Bench (LI-COR Inc., Lincoln, NE, USA). Maximum leaf light-saturated photosynthetic rate (*P*_max_) and light saturating point (LSP) were estimated.

### C_i_ Response Curves

To study the relationship between *P*_n_ and leaf internal CO_2_ concentration *C*_i_, photosynthesis was measured on two clear days, 75–76 DAP. Leaf temperature inside the leaf chamber was maintained at 20°C, and PPFD, at 1000 µmol photons m^–2^s^–1^. *P*_n_ was measured at *C*_a_ in the following order: 400, 250, 150, 100, 50, 0, 400, 400, 600, 800, 1000 and 1200 µmol mol^–1^ provided by a CO_2_ mixer. Sigma Plot for Windows 10.0 was used to fit the *P*_n_/*C*_i_ response curves using an exponential function [19]:
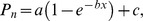
where *P*_n_ is leaf net photosynthetic rate and *x* is *C*_i_. Using this equation, the CO_2_ saturated rate of photosynthesis (*P*_sat_) was calculated as *a*+*c*, and the carboxylation efficiency (CE), as the slope at *P*_n_ = 0 or b(a+c).

Maximum carboxylation rate of Rubisco (*V*_cmax_) and maximum light-saturated rate of electron transport (*J*_max_) were determined using Photosynthesis Assistant software (Version 1.1, Dundee Scientific, Dundee, UK) according to Farquhar *et al.* [20], modified by Harley and Sharkey [21] and Harley *et al.* [22].

### Soluble Protein and Rubisco Contents

The leaves used for light and *C*_i_ response curves were collected to determine soluble protein and Rubisco content. Approximately 0.5 g of fresh leaf material per sample with the mid-vein removed was ground in liquid nitrogen to a fine powder with 10 mg of PVPP. Extraction buffer [50 mM sodium phosphate buffer pH 7.8, 10% (v/v) glycerol, 1% (v/v) β-mercaptoethanol] was added at 3 ml g^–1^ fresh weight. The homogenate was centrifuged at 16,000 g for 15 min at 4°C. Protein concentration of the supernatant was estimated by the protein dye-binding method of Bradford [23] using bovine serum albumin (BSA) as the standard.

Rubisco content was determined following the protocol of Makino *et al.* [24] modified by Irving and Robinson [25]. Briefly, equal amounts of protein and extraction buffer were mixed and boiled for 2 min. Proteins in the extracts together with bovine serum albumin standards were separated using SDS-PAGE following the method of Laemmli [26] using 12% acrylamide resolving and 5% acrylamide stacking gels and the Mini-PROTEAN 3 System (Bio-Rad Laboratories, Richmond, CA, USA). Gels were stained using 1% (w/v) Coomassie Brilliant Blue R250 for 3 hours, the Rubisco containing band was excised, and the protein concentration was determined spectrophotometrically at 595 nm after elution of the stain in formamide at 50°C for 12 hours.

### Carbon and Nitrogen Analysis

Tissue C and N concentrations in *M. micrantha* and in stems and flowers of *C. campestris* plants harvested on 90 DAP were assayed by GC using a Vario EL CHNS elemental analyzer (Elementar Analysensysteme GmbH, Hanau, Germany). They were also determined for the leaves used to measure photosynthesis. Photosynthetic nitrogen use efficiency (PNUE) was calculated as *P*_max_/*N*_area_.

### Data Analysis

All statistical tests were carried out at α = 0.05 level using SPSS (version 11.5, SPSS Inc., Chicago, IL, USA). Two-way analysis of variance (ANOVA) was performed to evaluate the effects of nitrate supply, *C. campestris* infection, and their interaction on the growth and physiological traits. Repeated measures ANOVA was conducted to test the main effects, their interactions and measuring times (0, 15, 40, 60 and 90 DAP) on the number of leaves. One-way ANOVA was performed to test the effects of nitrate treatments on parasite biomass. Treatment means of the significant ANOVA effects were compared at α = 0.05 level using the least significant difference (LSD) analysis. Correlation analysis was conducted to test the relationships between *P*_sat_ or CE and leaf nitrogen concentrations for *M. micrantha* plants. To satisfy the assumptions of ANOVA, some data were square-root transformed; however, untransformed data are presented in tables and figures.

## Results

There were no differences in the flowering initiation of the uninfected or infected *M. micrantha* among the three nitrate treatments. In both uninfected and infected plants, compared with 5 mM nitrate fertilization, the other two delayed the further development of inflorescence after the inflorescence had formed and such delay was more at 0.2 than at 1 mM nitrate (data not shown), and they also reduced the number of florets. At all nitrate levels, the uninfected started to develop terminal inflorescences on 15 DAP, but the infected, on 40 DAP. From 15 to 60 DAP, *C. campestris* grew vigorously with a lot of branching. It started flowering on 20 DAP at 0.2 mM, and on 25 DAP at 1 or 5 mM nitrate treatments.

### Number of Leaves

**Figure 1:**
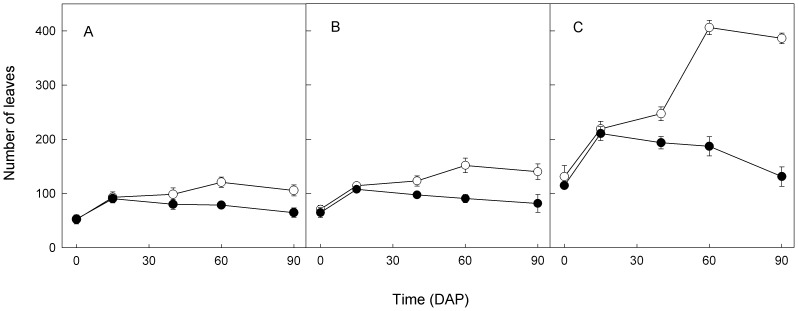
Means (±SE, *n* = 5) of number of leaves of uninfected (○) and infected (•) *M. micrantha* plants by *C. campestris* on different days after parasitization (DAP) at 0.2 (A), 1 (B) and 5 (C) mM nitrate fertilizations.

Repeated measures ANOVA indicated there were significant (*P*<0.001) infection, nitrate and their interaction effects on the number of leaves of *M. micrantha* over the measurement times. From 0 to 15 DAP, the number of leaves increased regardless of infection and fertilization treatments (Figure 1). From 15 to 90 DAP, the number of leaves of the infected *M. micrantha* was smaller than that of the control, and the differences between them became greater as nitrate fertilization levels increased from 0.2 to 1 to 5 mM. The number of leaves of the infected decreased continuously from 15 to 90 DAP, and that of the control increased from 0 to 60 DAP and then decreased slightly from 60 to 90 DAP. At harvest on 90 DAP, infected plants had 61%, 58% and 34% of the number of leaves of uninfected plants at 0.2, 1 and 5 mM nitrate supplies, respectively.

### Plant Biomass Components

**Figure 2:**
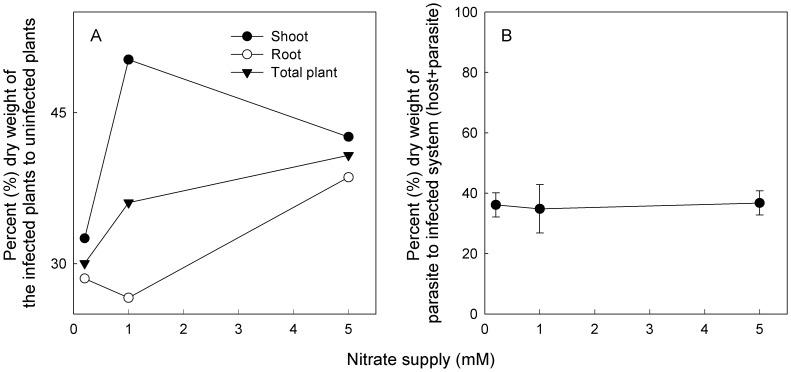
Percent (%) dry weight of the infected to the uninfected *M. micrantha* plants (A) and of the parasite to infected system (host plus parasite) (B) in the association *M. micrantha*-*C. campestris* fertilized at 0.2, 1 and 5 mM nitrate fertilizations.

By 90 DAP, the dry mass of the infected system (host plus parasite) was significantly less than that of uninfected *M. micrantha* across all nitrate treatments (Table 1). *Mikania micrantha* total biomass and its components were significantly reduced by *C. campestris* infection at all nitrate treatments, and the magnitude of the reduction was dependent on nitrate fertilization levels as indicated by significant nitrate × *Cuscuta* interaction (Table 1). The infection reduced *M. micrantha* root biomass by about 71%, 73% and 61%, flower biomass by about 91%, 79% and 71% and total biomass by about 70%, 64% and 59% at 0.2, 1 and 5 mM nitrate fertilizations, respectively. These proportional decreases in biomass with the increases in nitrate supply occurred although the infected plants supported significantly higher parasite biomass at high than at low nitrate fertilizations (Figure 2A; Table 1). However, the parasite was always a similar proportion of the infected system (host plus parasite) at all nitrate levels (Figure 2B).

**Table 1:** ANOVA results and means (±SE, *n* = 5) of plant dry biomass (g) components based on data collected on 90 DAP for uninfected and infected *M. micrantha* plants by *C. campestris* at 0.2, 1 and 5 mM nitrate fertilization concentrations.

	*M. micrantha*						
Treatments	Roots	Stem	Leaves	Flowers	Total	Infected system (host+parasite)	*C. campestris*
0.2 mM nitrate							
Uninfected	22.64±2.17c	8.50±0.76de	3.86±0.49c	1.17±0.50bd	36.16±3.25c	36.16±3.25d	
Infected	6.46±1.91d	2.66±0.41c	1.63±0.14d	0.11±0.11c	10.86±2.28d	16.44±2.12e	5.58±0.22b
1 mM nitrate							
Uninfected	32.95±3.37b	12.70±0.75bd	6.44±0.64b	2.88±0.53b	54.97±4.20b	54.97±4.20c	
Infected	8.77±1.78d	7.12±1.69ce	3.35±0.65cd	0.60±0.30cd	19.83±4.27d	29.04±3.23d	9.21±1.33b
5 mM nitrate							
Uninfected	53.10±3.51a	35.35±5.22a	14.94±0.86a	13.87±3.25a	117.26±5.61a	117.26±5.61a	
Infected	20.49±2.96c	17.06±2.69b	6.31±1.01b	3.96±1.32b	47.81±4.63bc	75.39±4.55b	27.57±2.82a
Source of variation	*F* values from ANOVA						
Nitrate (N)	35.89***	36.77***	70.12***	18.72***	109.36***	170.00***	42.76***†
Infection (I)	121.16***	22.86***	68.59***	13.55**	161.39***	80.36***	
N × I	4.61*	4.11*	12.77***	5.33*	15.42***	4.11*	

† F value from one-way ANOVA, and the rest *F* values are from two-way ANOVA.

ns, *P*>0.05; *P<0.05; **P<0.01, ***P<0.001. Means in the same column not sharing a common letter are significantly different according to LSD analysis at *p* = 0.05 level. The same apply to Tables 2, 3, 4, 5, 6 and 7.

### RGR and Leaf Area

RGR was affected significantly by nitrate and infection, but not by their interaction (Table 2). Significant decreases in RGR occurred in the infected *M. micrantha* plants at each nitrate fertilization level, and generally as nitrate supplies increased, RGR increased within each infection treatment. Infection significantly reduced leaf area of *M. micrantha*, and this negative effect was greater at 5 than at 0.2 or 1 mM nitrate (Table 2).

**Table 2:** ANOVA results and mean (±SE, *n* = 5) of relative growth rate (RGR, g g^–1^d^–1^), leaf area (m^2^), leaf area ratio (LAR, m^2^ kg^–1^ of plant, specific leaf area (SLA, m^2^ kg^–1^ of leaves), unit leaf rate (ULR, g m^–2^ d^–1^), shoot:root dry weight ratio (S:R, g g^–1^), and (host shoot+*Cuscuta*):host root ((H+C)/HR, g g^–1^) for *M. micrantha* plants infected and uninfected by *C. campestris* and fertilized at 0.2, 1 and 5 mM nitrate.

Treatments	RGR	Leaf area	LAR	SLA	ULR	S:R	(H+C)/HR
0.2 mM nitrate							
Uninfected	0.045±0.001b	0.12±0.02c	4.12±0.28c	32.00±0.93ab	11.22±0.78a	0.61±0.05c	0.61±0.05b
Infected	0.031±0.002d	0.06±0.01d	6.05±0.88ab	33.63±2.57ab	5.79±1.10b	0.87±0.20bc	2.03±0.47a
1 mM nitrate							
Uninfected	0.052±0.001a	0.18±0.02b	4.16±0.13c	29.04±1.78b	12.55±0.46a	0.69±0.05c	0.69±0.05b
Infected	0.040±0.002c	0.12±0.02c	6.65±0.67a	34.54±2.63ab	6.24±0.82b	1.24±0.11ab	2.63±0.44a
5 mM nitrate							
Uninfected	0.056±0.001a	0.45±0.03a	4.89±0.15bc	30.27±1.92ab	11.42±0.36a	1.22±0.05ab	1.22±.0.05b
Infected	0.046±0.001b	0.22±0.04b	5.80±0.48ab	35.06±1.88a	8.07±0.66b	1.41±0.22a	2.84±0.32a
Source of variation	*F* values from ANOVA						
Nitrate (N)	32.79***	50.31***	0.22 ns	0.15 ns	1.51 ns	9.28**	2.90 ns
Infection (I)	94.98***	34.23***	18.02***	5.75*	69.48***	9.27**	47.48***
N × I	0.80 ns	6.63**	1.24 ns	0.51 ns	2.11 ns	1.01 ns	0.39 ns

### Biomass Allocation

Biomass allocation parameters, except the percentage of total biomass allocated to flowers, of *M. micrantha* were all significantly affected by infection, but not by the interaction of nitrate and infection (Tables 2, 3).

**Table 3:** ANOVA results and means (±SE, *n* = 5) of the percentages (%) of total biomass allocated to roots, stems, leaves and flowers of the uninfected and infected *M. micrantha* plants by *C. campestris* on 90 DAP under 0.2, 1 and 5 mM nitrate fertilization treatments.

Treatments	Roots	Stems	Leaves	Flowers
0.2 mM nitrate				
Uninfected	62.55±2.34a	23.69±1.36b	10.66±0.74b	3.10±1.08b
Infected	55.93±5.58a	25.44±1.57b	17.11±3.18a	1.53±1.53b
1 mM nitrate				
Uninfected	59.47±1.82a	23.35±1.34b	11.73±0.84b	5.44±1.14bc
Infected	45.00±2.18b	35.27±0.82a	17.25±1.11a	2.48±1.19b
5 mM nitrate				
Uninfected	45.16±1.16b	29.86±3.24ab	12.74±0.45b	12.23±2.92a
Infected	42.81±3.85b	35.87±4.04a	12.93±1.08ab	8.38±1.93ac
Source of variation	*F* values from ANOVA			
Nitrate (N)	11.58***	6.21**	0.60 ns	9.14**
Infection (I)	9.10**	11.53**	10.57**	3.00 ns
N × I	1.88 ns	2.33 ns	2.45 ns	0.17 ns

Generally, *C. campestris* infection significantly increased LAR, SLA and shoot:root ratios (S:R), but it reduced ULR of *M. micrantha* plants, and its effects on these traits were independent of nitrate treatments (Table 2). Within each nitrate treatment, the infection effects were more negative on root than on shoot growth (Figure 2A), resulting in higher S:R in infected plants than in control plants. The ratio of above to below-ground biomass in the host-parasite system was 2.3–3.8 times that of the uninfected plants among the nitrate treatments (Table 2).

*Cuscuta campestris* infection increased biomass allocation to stems and leaves and reduced them to roots and flowers of *M. micrantha* plants although the effects within all nitrate levels were not always significant (Table 3). The interaction of nitrate and infection was not significant in the allocations to these biomass components of *M. micrantha* plants.

### P_n_ of *M. micrantha* Leaves

The interaction of nitrate and infection had no significant effects on *P_n_* and related parameters of *M. micrantha* leaves on 30 and 80 DAP (Table 4). The infected plants had lower leaf *P_n_*, *g*_s_, *E* and WUE, but higher *C*_i_ than the uninfected plants at each nitrate fertilization level. Mostly, nitrate treatment did not result in significant changes in *P*_n_, *g*_s_, *E*, *C*_i_ and WUE measured on 80 DAP but led to no consistent changes in these traits on 30 DAP within infection treatments (Table 4).

**Table 4:** ANOVA results and means (±SE, *n* = 5) of the net photosynthetic rate (*P*_n_), stomatal conductance (*g*_s_), transpiration rate (*E*), water use efficiency (WUE), intercellular CO_2_ concentration (*C*_i_) of the youngest fully expanded mature leaves of the uninfected and infected *M. micrantha* plants by *C. campestris* on different days after parasitization (DAP) under different nitrate fertilization treatments.

	*P*_n_ (µmol CO_2_ m^–2^ s^–1^)		*g*_s_ (mol H_2_O m^–2^s^–1^)		*E* (mmol H_2_O m^–2^s^–1^)		*C*_i_ (µmol mol^–1^)		WUE (µmol CO_2_ mmol ^−1^ H_2_O)	
Treatments	30 DAP	80 DAP	30 DAP	80 DAP	30 DAP	80 DAP	30 DAP	80 DAP	30 DAP	80 DAP
0.2 mM nitrate										
Uninfected	5.15±0.42bd	4.47±0.42a	0.11±0.01bc	0.09±0.02a	1.64±0.15b	1.10±0.21ab	278.52±4.67ab	259.68±6.96bc	3.16±0.14b	4.38±0.45b
Infected	2.23±0.28c	1.75±0.34b	0.09±0.02c	0.07±0.02ab	1.53±0.37b	0.71±0.13bc	303.14±8.16a	316.51±8.26a	1.65±0.30ac	2.48±0.27c
1 mM nitrate										
Uninfected	6.22±0.85b	4.07±0.54a	0.16±0.03a	0.06±0.01ab	1.48±0.19b	0.71±0.13ac	287.72±8.21a	240.37±8.87c	4.30±0.45a	5.99±0.43ab
Infected	4.08±0.35cd	1.08±0.27b	0.12±0.01ac	0.04±0.01b	1.46±0.17b	0.46±0.11c	290.68±12.95a	289.95±21.27ab	3.04±0.55bc	2.50±0.72c
5 mM nitrate										
Uninfected	8.44±0.97a	4.48±0.66a	0.15±0.03ab	0.07±0.01ab	2.71±0.42a	0.73±0.14ac	255.01±12.06b	231.18±13.66c	3.28±0.38ab	6.41±0.84a
Infected	3.51±0.85cd	1.21±0.58b	0.09±0.02c	0.04±0.01b	1.66±0.30b	0.40±0.11c	289.19±6.47a	295.72±11.37ab	2.09±0.24c	2.59±0.53c
Source of variation	*F* values from ANOVA									
Nitrate (N)	5.80**	0.60 ns	3.57*	2.71 ns	3.59*	3.55*	2.53 ns	2.36 ns	6.51**	1.92 ns
Infection (I)	36.11***	56.44***	7.85*	4.51*	2.83 ns	7.79*	7.46*	30.33***	19.31***	43.23***
N × I	2.26 ns	0.16 ns	0.43 ns	0.11 ns	1.96 ns	0.14 ns	1.50 ns	0.17 ns	0.10 ns	1.62 ns

### Photosynthesis in Response to Light

*Cuscuta campestris* infection had significant effects on LSP, *P*_max_, LCP and Φ, but not on *R*_d_ of *M. micrantha* leaves in response to light (Figure S1; Table 5). Leaves of uninfected plants had higher LSP, *P*_max_ and Φ but lower LCP than infected plants at each nitrate treatment. However, nitrate and its interaction with infection had no significant effects on these parameters (Table 5).

**Table 5:** ANOVA results and means (±SE, *n* = 5) of photosynthesis parameter estimates from the light response curves for the youngest fully expanded mature leaves of the uninfected and infected *M. micrantha* by *C. campestris* under different nitrate fertilization concentrations.

	Parameter estimates of photosynthesis					
Treatments	LSP (µmol photonsm^–2^ s^–1^)	*P*_max_ (µmol CO_2_m^–2^ s^–1^)	LCP (µmol photonsm^–2^ s^–1^)		Φ (mol CO_2_ mol^–1^ photons)	*R*_d_ (µmol m^–2^ s^–1^)
0.2 mM nitrate						
Uninfected	376.00±53.11bc	4.96±0.41a	9.73±0.73c		0.042±0.002a	0.40±0.03
Infected	293.60±23.38c	2.19±0.43b	21.09±3.84ab		0.021±0.004b	0.40±0.07
1 mM nitrate						
Uninfected	516.00±65.62ab	5.17±0.70a	16.16±3.43abc		0.042±0.005a	0.62±0.07
Infected	361.40±38.89bc	2.79±0.48b	25.39±2.80ab		0.015±0.001b	0.39±0.06
5 mM nitrate						
Uninfected	586.40±75.82a	5.33±0.77a	14.07±2.66bc		0.034±0.001a	0.46±0.08
Infected	273.40±33.00c	2.24±0.52b	27.96±5.45a		0.019±0.004b	0.42±0.04
Source of variation	*F* values from ANOVA					
Nitrate (N)	2.49 ns	0.26 ns	1.68 ns	0.83 ns		1.43 ns
Infection (I)	18.88***	35.10***	16.61***	45.17***		2.99 ns
N × I	2.61 ns	0.20 ns	0.23 ns	1.20 ns		1.83 ns

LSP, light saturation point; *P*_max_, net photosynthesis at LSP; LCP, light compensation point; Φ, apparent quantum yield; *R*_d_, dark respiration rate.

### Photosynthesis in Response to C_i_

Leaves of uninfected plants had significantly higher CE, *P*_sat_, *V*_cmax_ and *J*_max_ than infected plants at each nitrate treatment (Figure S2; Table 6). CE and *P*_sat_ were higher at 5 than at 0.2 and 1.0 mM nitrate in both the infected and uninfected.

**Table 6:** ANOVA results and means (±SE, *n* = 5) of the photosynthesis parameter estimates from the *P*_n_-*C*_i_ response curves for the youngest fully expanded mature leaves of the uninfected and infected *M. micrantha* by *C. campestris* under different concentrations of nitrate fertilization.

	Parameter estimates of photosynthesis			
Treatments	CE	*P*_sat_ (µmol CO_2_ m^–2^ s^–1^)	*V*_cmax_ (µmol m^–2^ s^–1^)	*J*_max_ (µmol m^–2^ s^–1^)
0.2 mM nitrate				
Uninfected	0.038±0.005b	8.22±0.47a	18.8±1.08ab	78.64±4.97ab
Infected	0.010±0.001cd	3.24±0.38bc	8.63±0.87c	36.02±3.54cd
1 mM nitrate				
Uninfected	0.034±0.006b	8.66±1.02a	19.6±2.71ab	87.84±13.98ab
Infected	0.006±0.001c	2.15±0.13c	6.76±0.78c	28.78±2.70d
5 mM nitrate				
Uninfected	0.061±0.008a	10.52±1.18a	23.5±2.14a	104.26±10.64a
Infected	0.025±0.009bd	4.87±1.05b	13.39±4.21bc	58.94±18.79bcd
Source of variation	*F* values from ANOVA			
Nitrate (N)	8.06**	4.70*	3.13 ns	3.21 ns
Infection (I)	38.53***	74.95***	34.07***	30.60***
N × I	0.27 ns	0.45 ns	0.23 ns	0.33 ns

CE, carboxylation efficiency; *P*_sat_, CO_2_ saturated rate of photosynthesis; *V*_cmax_, maximum rate of Rubisco carboxylase activity; *J*_max_, maximum rate of photosynthetic electron transport.

### Chlorophyll and Carotenoid

The concentrations of total chlorophyll, chlorophyll *a* and *b*, and carotenoid of *M. micrantha* leaves were significantly affected by nitrate, infection and their interaction (Table 7). There was a greater reduction in chlorophyll concentration of infected plants at 5 mM nitrate than at 0.2 or 1 mM nitrate (Table 7). The chlorophyll *a*:*b* ratio was not significantly affected by infection at each nitrate treatment.

**Table 7:** ANOVA results and mean (±SE, *n* = 5) concentrations (mg g^–1^) of total chlorophyll (Chl), Chl *a*, Chl *b*, carotenoid (mg g^–1^), Chl *a*:*b* ratio, soluble protein, Rubisco and nitrogen (g m^–2^) of the youngest fully expanded mature leaves of uninfected and infected *M. micrantha* plants fertilized at 0.2, 1 and 5 mM nitrate concentrations.

Treatments	Total Chl	Chl *a*	Chl *b*	Chl *a*/*b* ratio	Carotenoid	Soluble protein	Rubisco	Nitrogen	PNUE
0.2 mM nitrate									
Uninfected	0.73±0.04b	0.52±0.03b	0.21±0.01b	2.51±0.05b	0.057±0.003b	3.39±0.22b	1.08±0.14a	0.36±0.03c	12.73±1.56a
Infected	0.29±0.05c	0.20±0.04c	0.09±0.01c	2.25±0.20b	0.038±0.005c	1.27±0.17c	0.59±0.14b	0.36±0.06c	5.21±1.01bcd
1 mM nitrate									
Uninfected	0.70±0.12b	0.50±0.08b	0.20±0.03b	2.44±0.12b	0.055±0.009b	4.50±0.19a	1.42±0.13a	0.55±0.07b	7.58±1.01b
Infected	0.22±0.04c	0.15±0.03c	0.07±0.01c	2.25±0.17b	0.031±0.006c	1.41±0.41c	0.52±0.13b	0.31±0.01c	3.54±0.89cd
5 mM nitrate									
Uninfected	1.47±0.15a	1.10±0.11a	0.37±0.040a	2.97±0.04a	0.105±0.008a	5.12±0.42a	1.53±0.14a	0.76±0.06a	6.00±1.09bc
Infected	0.35±0.07c	0.26±0.05c	0.09±0.02c	3.10±0.23a	0.046±0.007bc	2.08±0.44c	0.50±0.24b	0.49±0.03b	2.52±0.82d
Source of variation	*F* values from ANOVA								
Nitrate (N)	15.61***	18.02***	9.33**	12.92***	13.67***	7.38**	0.71 ns	16.55***	9.94**
Infection (I)	88.26***	87.35***	87.06***	0.75 ns	37.70***	104.26***	38.34***	19.58***	31.65***
N × I	9.38**	9.98**	7.48**	0.93 ns	5.28*	1.35 ns	1.61 ns	5.07*	2.01 ns

### Proteins and Rubisco Contents

Nitrate treatment had a significant influence on total soluble protein content, but not on Rubisco content (Table 7). Higher nitrate supply resulted in higher soluble protein content. Infection significantly reduced both total soluble protein and Rubisco contents, and the response to infection was similar across nitrate levels (Table 7).

### Leaf Nitrogen, Nitrogen Partitioning and PNUE

**Figure 3:**
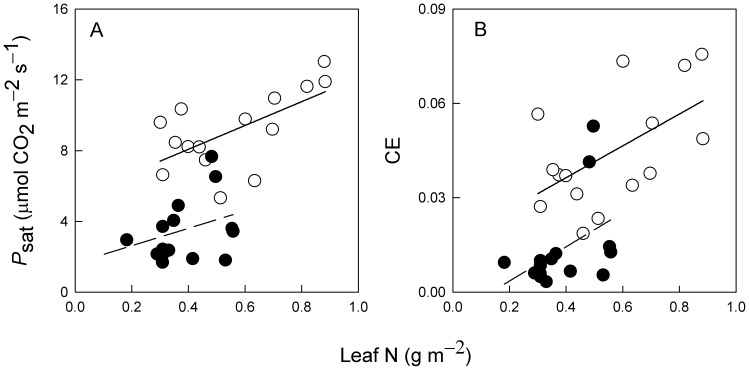
The relationships between leaf nitrogen concentrations (Leaf N) and *P*_sat_ (A) and CE (B) for *M. micrantha* plants either uninfected (○; solid line) or infected (•; broken line) with *C. campestris* (Data from all nitrate treatments are included). The correlation coefficients are 0.62 (*P*<0.05) and 0.40 (*P*>0.05) in (a), and 0.56 (*P*<0.05) and 0.44 (*P*>0.05) in (b) for the uninfected and infected plants, respectively.

*Cuscuta campestris* infection significantly reduced *M. micrantha* plant leaf nitrogen content and its effect depended on nitrate supply (Table 7). The infected plants had significantly reduced leaf nitrogen contents at 1 and 5 mM nitrate fertilizations, but not at 0.2 mM. The nitrogen concentrations in *C. campestris* were not significantly different among the three nitrate fertilizations; 14.4±0.66, 15.2±0.76 and 16.9±0.60 mg g^–1^ at 0.2, 1 and 5 mM nitrate, respectively. There was a significant positive linear correlation between *P*_sat_ or CE and leaf nitrogen content for uninfected *M. micrantha* plants (Figure 3). *Cuscuta campestris* infection significantly reduced photosynthetic nitrogen use efficiency (PNUE) of *M. micrantha* plants but the negative effects did not differ across the three nitrate treatments (Table 7).

## Discussion

The present study shows that *C. campestris* infection had significant effects on most of the traits related to growth (biomass traits, number of leaves and leaf area), biomass allocation, photosynthesis and biochemical parameters of *M. micrantha* host plants. The extent of the negative effects of the parasite on host growth, and chlorophyll and leaf nitrogen content varied with the concentration of nitrate supplied to the host plants, as indicated by the significant nitrate × infection effects on these variables. However, the effects of infection on biomass allocation and leaf photosynthesis related traits of *M. micrantha* were independent of nitrate supply.

### Growth

In the present study, *C. campestris* infection reduced the number of leaves, leaf area, and biomass traits (total, root, stem, leaf and flower dry weights) of *M. micrantha* plants at each level of nitrate fertilization, and the negative impacts of the parasite on host growth were generally less severe at high than at low nitrate supplies. Such an alleviation of the impacts at high nitrogen was attributable to a more negative influence of the infection on root biomass at low than at high nitrogen fertilization. Alleviation of growth inhibition at high N supply has also been observed in *R. communis* infected by *C. reflexa* [8] and in sorghum [5] or rice [6] infected by *S. hermonthica*. However, as N supply increased, inhibition in the growth of *C. blumei* infected by *C. reflexa* increased [7], which might be due to the strangling effect exerted by the haustorial coil of *C. reflexa*. Such strangling was not found in *C. reflexa*-infected *R. communis* [8] or in *C. campestris*-infected *M. micrantha* in this study.

Inhibitions in the reproductive growth of infected plants also became more severe at lower nitrate supply with fewer resources for reproduction as growth became severely inhibited. It has been reported that flowering was delayed and the number of florets was reduced at high N supply in *C. reflexa*-infected *C. blumei* [7], *R. communis* [8], *Vicia faba* [27] and *L. albus* [9], and flowering of *M. micrantha* was completely inhibited by low N supply and by *C. campestris* infection [10]. We did not find complete flowering inhibition in the present study which contradicts results of our earlier study [10]. However, although the plants in both studies were of similar age at the time of infection treatments, the treatments were applied about 45 days later in the growing season in the present than in the previous study.

In this study, the total biomass of the infected system (host+parasite) was less than uninfected *M. micrantha*, with greater proportional reductions as nitrate concentration decreased. Similar results were observed in the same system [10] and in *S. hermonthica*-sorghum association [5]. In these cases, the reduced growth of infected plants resulted from resource capture by the parasite and the negative effects of the parasite on host photosynthesis. As infection reduced host photosynthesis in our present study at each level of nitrate supplies, and in our previous study [11], the response of the host to infection cannot be explained by a simple source-sink relation regardless of nitrogen treatment [1], [7], [8]. As nitrate supply increased, the biomass of infected hosts increased, as did the corresponding biomass of the parasite. However, the percentage of total biomass allocated to the parasite did not differ among the three nitrate treatments, indicating the growth of the parasite is dependent on or tuned to the size or carrying capacity of the host. Therefore, *C. campestris* growth on its host may be resource-dependent: resource uptake is not linear but eventually reaches a plateau. This is possible as *C. campestris* obtains all its resources from its host *M. micrantha*, and its host’s physiological conditions would change directly with parasite densities; in turn, *C. campestris* somehow ‘senses’ these changes and then regulates its growth accordingly. A fine tuning of the sink power of the parasite in the association *R. communis*–*C. reflexa* [8] and the adaptation of *C. campestris* life cycle completion to the resource availability of its host *M. micrantha* [10] has been observed. Such sensing or tuning strategies can ensure the survival of the hosts for the normal growth and development of the parasites, and the biochemical and physiological mechanisms underlying them are unknown and merit future studies.

### Biomass Allocation

*Cuscuta campestris* had more negative effects on host root than shoot growth. *Cuscuta campestris* infection resulted in greater biomass allocation to stems and leaves but lesser allocation to roots, thus resulting in increased shoot:root ratios of infected *M. micrantha* plants. Similar results were found in our previous studies [10], [28]. *Cuscuta campestris* is a shoot parasite and competes for the resources that the host allocates to shoot and root growth. The host may allocate relatively more resources to shoots to compensate for the resources directly captured by the parasite, or transfer relatively fewer resources to roots, or a higher competitive demand and a stronger source demand from the shoot system resulting in greater transfer of resources to shoots. In root parasites, reduced shoot:root ratios have been reported in *S. hermonthica*-infected rice and sorghum [5], [6] and *Orobanche aegyptiaca*-infected tomato [29]. Therefore, the negative effects of shoot parasites may be more severe on the roots than on the shoots of their hosts, and the opposite may apply to root parasites. This requires further study.

### Photosynthesis

Our previous studies [11], [30] showed that *C. campestris* infection reduced leaf *P*_n_ of *M. micrantha* and speculated that this was due to the parasite’s indirect adverse impacts on *g*_s_ and direct negative effects on the photosynthetic metabolism of *M. micrantha*. Our present study shows similar negative effects resulting from the lower light and CO_2_ use efficiencies of leaves of infected plants than uninfected plants. Infection reduced LSP, *P*_max_, Φ, *P*_sat_, CE, *V*_cmax_ *J*_max_ and PNUE and increased LCP across all nitrate levels. Lower photosynthetic efficiency of infected plants was also caused by lower leaf nitrogen, chlorophyll *a* and *b*, soluble protein and Rubisco concentrations, and *g*_s_ than uninfected plants. Low nitrogen concentrations in infected leaves could accelerate leaf senescence, reducing leaf photosynthesis and the number of leaves. This chain of effects in infected plants explains the lower photosynthesis we observed at leaf level, resulting in lower total photosynthesis and growth at the plant level in comparison to uninfected plants.

The magnitude of the negative effects of *C. campestris* on *M. micrantha* photosynthesis was similar across all nitrate fertilization levels. Thus, the less severe inhibition in host growth at high than at low nitrate levels is not attributable to inhibition of host photosynthesis and hence leaf production. It has been shown that *Cuscuta* can form a strong sink to redirect the flow of host resources to itself [8], [27], and *Cuscuta* species alter host physiology by acting as a stronger sink for photosynthates than any host organs [31]. Redirection of more resources by *C. campestris* at low than at high nitrate levels resulted in a greater reduction in infected *M. micrantha* total biomass and root biomass.

Uninfected *M. micrantha* plants made greater use of the extra nitrogen to produce ‘greener’ leaves than infected plants, as shown by the higher leaf chlorophyll content at 5 mM than at 1 and 0.2 mM nitrate in uninfected plants. However, greener leaves did not have increased *P*_max_ although there was a good relationship between N concentration and *P*_sat_. The significant effect of nitrate and infection interaction on leaf chlorophyll content (per leaf area or leaf mass) resulted from the chlorophyll content of the leaves of infected plants being more reduced at 5 mM than at 0.2 or 1 mM nitrate. This was consistent with the variation pattern for leaf nitrogen content per unit leaf area. The reason for this might be that the total nitrogen absorption and supply capacities of the roots of infected plants were more reduced at high than at low nitrate level.

Infection reduced transpiration and *g*_s_ on 30 and 80 DAP at each nitrate treatment, which may have induced stomatal closure or reduced stomatal opening. Parallel reductions in leaf nitrogen and *P*_n_ were observed, and *P*_n_ decreases would reduce carbon production. It has been suggested that low leaf nitrogen often leads to high leaf abscisic acid (ABA) levels and increases in xylem translocation of ABA from root to shoot [32] and high leaf ABA induces stomatal closure [30]. *Cuscuta campestris* infection lowered leaf nitrogen in *M. micrantha*, which may increase leaf ABA and thus contribute to the stomatal closure of infected plants [30].

In this study, as in our previous studies, *C. campestris* reduced the number of leaves of *M. micrantha*, through a host response of reducing new leaf initiation and/or accelerating leaf senescence and abscission [10], [11]. Leaf senescence is characterized by a decline in photosynthesis accompanied by the loss of Rubisco and chlorophyll/protein complexes and the decline in stomatal conductance [33]–[35]. Leaf chlorophyll and protein contents are often used as indicators of leaf senescence [36]. In the present study, the lower leaf *P*_n_ and *g*_s_, leaf nitrogen, total soluble protein and chlorophyll concentrations in infected plants than in uninfected plants suggest that host leaf senescence is a response to *C. campestris* infection.

In summary, the results indicate that the negative effects of the holoparasite *C. campestris* on the growth of *M. micrantha* were dependent on nitrate supply to the host, and they were more severe at low than at high nitrate levels. The more severe inhibition in host growth at low than at high nitrate supplies is largely attributable to the transfer of more host resources to *C. campestris* at low than at high nitrate levels as the magnitude of inhibition in host photosynthesis was similar across nitrate levels. In addition, *C. campestris* seems able to sense the carrying capacity of the host and regulates its growth accordingly, indicating a synchronicity in growth and development between the parasite and its host.

## Supporting Information

Figure S1 Mean net photosynthetic rates (*P*_n_, ±SE, *n* = 5) at different photosynthetic photon flux densities (PPFD) for the youngest fully expanded mature leaves of the uninfected (○) and infected (•) *M. micrantha* plants by *C. campestris* at (a) 0.2, (b) 1 and (c) 5 mM nitrate fertilizations.
(TIF)

Figure S2 Response of net photosynthetic rates (*P*_n_) to intercellular CO_2_ concentrations (*C*_i_) in the youngest fully expanded mature leaves of the uninfected (○) and infected (•) *M. micrantha* plants by *C. campestris* at (a) 0.2, (b) 1 and (c) 5 mM nitrate fertilizations. Data points are means ±SE (*n* = 5).
(TIF)
