# Supplementary figures and images for: Growth but Not Photosynthesis Response of a Host Plant to Infection by a Holoparasitic Plant Depends on Nitrogen Supply
Source: PLoS One. 2013 Oct 7;8(10):e75555. doi: 10.1371/journal.pone.0075555 (PMC3792126; doi:10.1371/journal.pone.0075555)

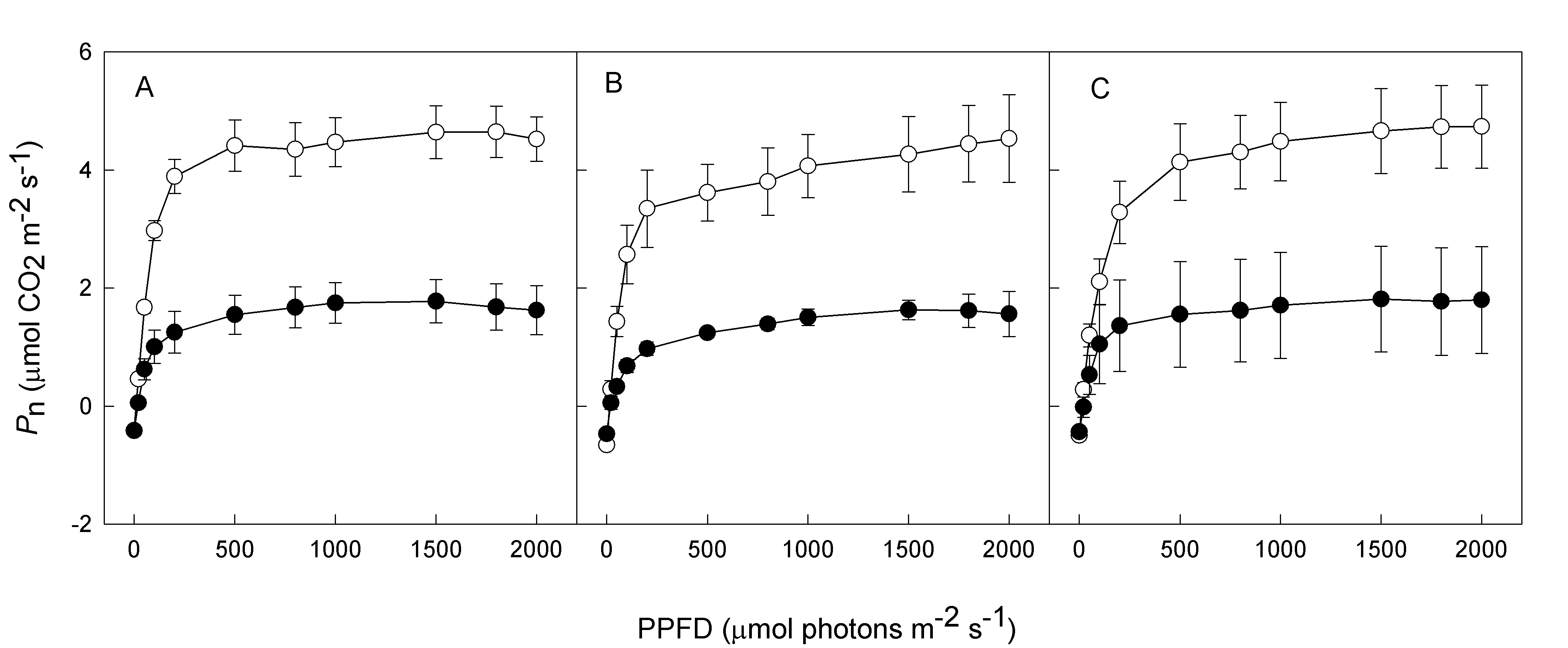

Supplement: Figure S1 — Mean net photosynthetic rates (P n, ±SE, n = 5) at different photosynthetic photon flux densities (PPFD) for the youngest fully expanded mature leaves of the uninfected (○) and infected (•) M. micrantha plants by C. campestris at (a) 0.2, (b) 1 and (c) 5 mM nitrate fertilizations. (TIF) [file pone.0075555.s001.tif]

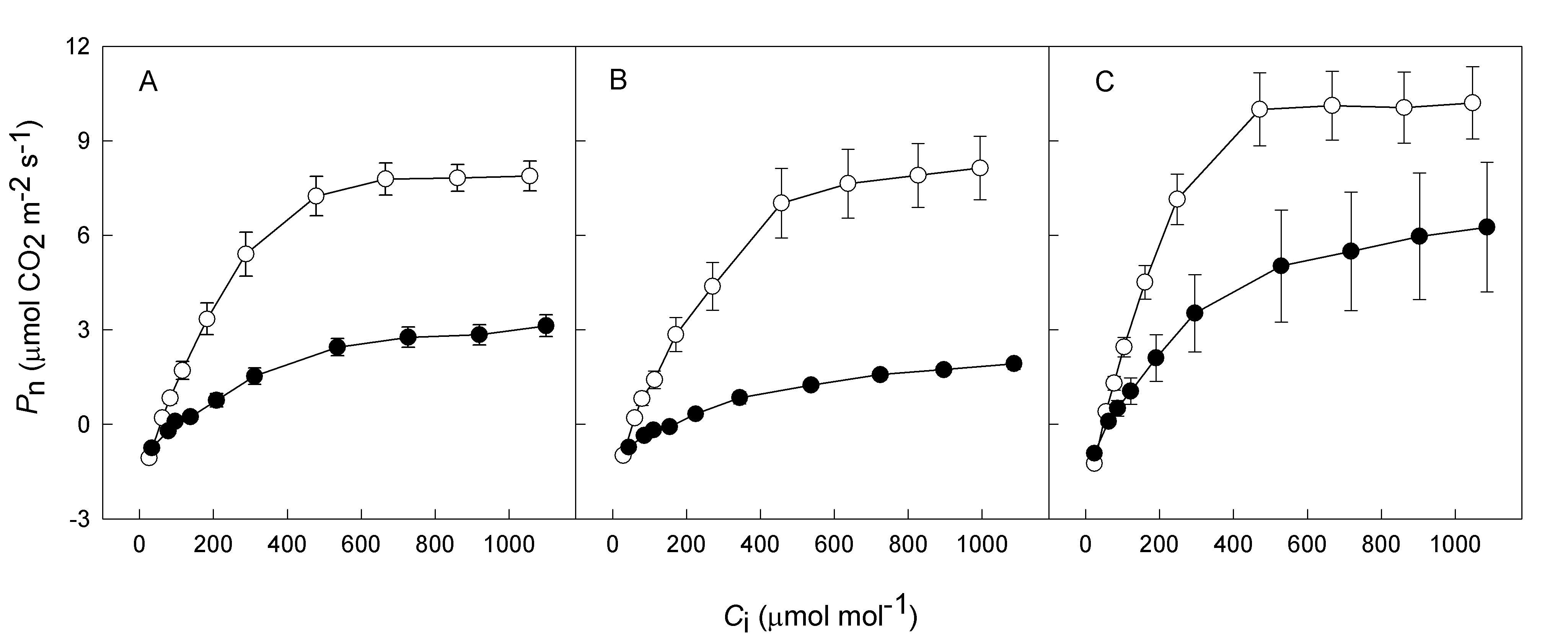

Supplement: Figure S2 — Response of net photosynthetic rates (P n) to intercellular CO2 concentrations (C i) in the youngest fully expanded mature leaves of the uninfected (○) and infected (•) M. micrantha plants by C. campestris at (a) 0.2, (b) 1 and (c) 5 mM nitrate fertilizations. Data points are means ±SE (n = 5). (TIF) [file pone.0075555.s002.tif]
